# Evaluating a cross-lagged panel model between problematic internet use and psychological distress and cross-level mediation of school administrator support on problematic internet use: The serial mediating role of psychological needs thwarting of online teaching and psychological distress

**DOI:** 10.3389/fpubh.2022.987366

**Published:** 2022-11-02

**Authors:** I-Hua Chen, Hsin-Pao Chen, Jeffrey H. Gamble, Xiao ling Liao, Xiu-Mei Chen, Ya-Ting Carolyn Yang, Amir H. Pakpour, Mark D. Griffiths, Chung-Ying Lin

**Affiliations:** ^1^Chinese Academy of Education Big Data, Qufu Normal University, Qufu, China; ^2^Division of Colon and Rectal Surgery, Department of Surgery, E-Da Hospital, School of Medicine, College of Medicine, I-Shou University, Kaohsiung, Taiwan; ^3^Department of English, National Changhua University of Education, Changhua, Taiwan; ^4^International College, Krirk University, Bangkok, Thailand; ^5^Faculty of Education, Qufu Normal University, Qufu, China; ^6^Institute of Education, National Cheng Kung University, Tainan, Taiwan; ^7^Department of Nursing, School of Health and Welfare, Jönköping University, Jönköping, Sweden; ^8^International Gaming Research Unit, Psychology Department, Nottingham Trent University, Nottingham, United Kingdom; ^9^Institute of Allied Health Sciences, College of Medicine, National Cheng Kung University, Tainan, Taiwan; ^10^Department of Occupational Therapy, College of Medicine, National Cheng Kung University, Tainan, Taiwan; ^11^Biostatistics Consulting Center, National Cheng Kung University Hospital, College of Medicine, National Cheng Kung University, Tainan, Taiwan; ^12^Department of Public Health, College of Medicine, National Cheng Kung University, Tainan, Taiwan

**Keywords:** administrator support, problematic internet use, problematic gaming, problematic social media use, psychological distress, psychological needs thwarting, teacher

## Abstract

**Background:**

To reduce the transmission of COVID-19, many teachers across the globe, including teachers in China, were required to teach online. This shift to online teaching can easily result in psychological need thwarting (PNT) of teachers' psychological basic needs (autonomy, competence, and relatedness), leaving them vulnerable to negative psychological outcomes. Resulting negative emotional state may lead to problematic internet use (PIU), which can lead to further psychological distress, forming a vicious cycle.

**Methods:**

The present study was conducted using a cross-lagged panel model (with longitudinal data) and hierarchical linear modeling (HLM) (with cross-sectional data). The aims were to investigate (i) the reciprocal relationships between two specific forms of PIU [problematic social media use (PSMU) and problematic gaming (PG)] and psychological distress among schoolteachers, and (ii) the influence of administrators' support on schoolteachers' PIU through a cross-level serial mediation model (PNT of online teaching was the first mediator and psychological distress was the second mediator affected by PNT of online teaching). Primary and secondary schoolteachers (*N* = 980; mean age = 34.76; 82.90% females) participated in two surveys (Time 1: mid-November 2021; Time 2: early-January 2022).

**Results:**

Results indicated that (i) high psychological distress at Time 1 was associated with increased levels of PSMU and PG at Time 2. Inversely, PG at Time 1 was associated with increased psychological distress at Time 2, although PSMU at Time 1 did not have a significant influence on psychological distress at Time 2; (ii) during Time 1, increased administrative support contributed to alleviating teachers' psychological needs thwarting of online teaching, thereby lowering their psychological distress which, in turn, resulted in a decrease in PG.

**Conclusion:**

PG had a stronger negative influence on teachers' psychological distress than PSMU. To relieve teachers' PG, administrative support can alleviate teachers' psychological needs thwarting of online teaching and psychological distress. Based on this finding, school managers must consider effective ways to support teachers during mandatory online teaching.

## Introduction

### Background

The COVID-19 pandemic has led to unprecedented changes in people's lives, with many different measures implemented to inhibit the spread of the disease. One widespread measure adopted worldwide was school closure which led to a shift from face-to-face teaching to online teaching (1, 2). Facing new job challenges during this period, the issue of schoolteachers' wellbeing was of great concern to both researchers and practitioners (1, 3–6). Not only did long periods of quarantine lead to an increase in negative psychological outcomes among many individuals (7, 8), but mandatory online teaching also contributed to additional work burden, stress, and mental health issues, particularly for schoolteachers with little experience with online teaching (9–12). Two systematic reviews carried out during the global COVID-19 outbreak between 2020 and 2021 further confirmed that a relatively high proportion of teachers suffered from mental health issues, with prevalence rates among teachers from Jordan, Brazil, USA, India, China, and Spain of (i) 15.9–28.9% for depression, (ii) 10–49.4% for anxiety, and (iii) 12.6–50.6% for stress (13, 14).

In addition to an increased work load due to the need to prepare online learning tasks (15–17), other sources of harm related to schoolteachers' mental health may have stemmed from other possible risk factors during this emergency period. One of these factors is problematic internet use (PIU), which has been shown to have a reciprocal relationship with negative psychological outcomes (i.e., each variable increases the vulnerability of experiencing the other) based on both cross-sectional and longitudinal studies (18, 19). A few studies have also found clear associations between PIU and teachers' negative psychological outcomes (9, 20, 21), including significant correlations with problematic social media use (PSMU) (*r* = 0.31) (9), significant differences between quartiles for emotional exhaustion, depersonalization, and decline of personal accomplishment (one-way ANOVA; *p* < 0.001) with higher internet addiction scores associated with higher levels of teacher burnout (21), and significantly higher frequency of depression among internet addicts vs. non addicts (*t*-tests; *p* < 0.001) (21). The worry concerning schoolteachers' PIU is reasonable in the context of COVID-19 because PIU often develops from excessive internet use (22, 23). In addressing the psychological pressure caused by the pandemic, consensus guidance by experts has noted that it is common for people to overuse information and communications technology (ICT) as a coping strategy and that some groups of individuals are at higher risk of developing problematic use patterns (24, 25). Given that it is difficult for teachers to avoid excessive use of ICT due to being restricted from turning off or muting ICT notifications due to work requirements (15–17), it is reasonable to assume that some schoolteachers might have developed PIU during the pandemic.

Moreover, the period during which schoolteachers encountered high levels of thwarting of competence, autonomy, and relatedness due to online teaching requirements may have also contributed to and/or caused mental health issues among schoolteachers. As a result of their unfamiliarity with technology for online teaching, teachers have commonly expressed frustration with their own competence (i.e., competence thwarting) (11, 12, 26). In fact, schoolteachers had to conduct online teaching following the rules enforced by governments, which may have negatively impacted teachers' sense of autonomy (i.e., autonomy thwarting) (27, 28). Additionally, in a study conducted by Weißenfels et al. (6), the emotional burden caused by online teaching, not the amount of work overload, was found to have a significant and negative influence on teachers' mental health during the COVID-19 pandemic. Their study showed that it was unfamiliarity with the mandatory tasks teachers faced rather than the amount of work that negatively impacted their mental health, suggesting teachers specifically require additional emotional support during online teaching. However, due to home confinement, teachers who lacked social interaction received limited verbal and non-verbal feedback from colleagues and administrators (28). This made it challenging for teachers to fulfill their increased emotional relatedness needs, resulting in relatedness thwarting when implementing online teaching. The thwarting of competence, autonomy, and relatedness together constitutes the construct of psychological needs thwarting (PNT) (29, 30). Based on scores using the Psychological Need Thwarting Scale of Online Teaching (PNTSOT), research has demonstrated high levels of schoolteachers' psychological need thwarting due to enforced online teaching (9). Such thwarting of basic psychological needs serves as a proxy for psychological ill-being (29–31), as opposed to wellbeing, potentially leading to mental health problems for teachers.

As noted above, teachers' competence, autonomy, and relatedness have been thwarted by the implementation of online teaching that has been lacking in the requisite material, emotional, and training support. This, notably, is the responsibility of administrators and, as such raises the issue of administrative support as an important variable for consideration in the context of online teaching. In order to tackle thwarting of teachers' competence, responses from American high school teachers have highlighted necessary administrative support in several areas, such as providing tools and related professional development, counseling, support, tips, and resources, including those which can improve the interactions among teachers, students and parents (32). In terms of autonomy, several studies have reported the impact of lack of administrative support on reduced work autonomy among teachers (33) while advocating for specific administrative actions to enhance autonomy, including clear curriculum guidelines for online teaching, provision of appropriate online platforms, scheduling of “self-care” time during the day, surveying of teachers to assess their needs, and expressing interest in teachers' input. In terms of relatedness, administrative support is a protective factor in terms of burnout, with emotional support, monitoring, provision of mental health to promote self-care, support for parent-teacher communication, and top-down guidance critical to the prevention of relatedness thwarting (34). Given the importance of administrative support, this variable will be evaluated in terms of reducing teachers PNT of online teaching which, in turn, can reduce psychological distress and related PIU behaviors. The purpose of this study will be clarified in the following sub-section.

### Purpose of the research

At present, in the context of the ongoing COVID-19 pandemic, studies have already confirmed the relationship between PIU and three aspects of PNT (i.e., autonomy, competence, and relatedness thwarting) of online teaching, and their contribution to negative psychological outcomes among teachers (9, 35). However, as far as we are aware, none of these studies have extended the scope of inquiry to examine the lasting effects of PIU, particularly during the onset of the pandemic, on teachers' subsequent mental health and potential bidirectional relationships (i.e., between psychological distress and PIU). Additionally, previous research has not examined how school administration influences teachers' psychological distress, PNT, and PIU. As such, the mechanisms relating to these variables remain largely unclear.

To bridge this gap, the present study conducted a two-wave survey (i.e., with the first wave of data collected during campus closure when online teaching was required and the second wave of data collected after campuses reopened and face-to-face classroom teaching resumed). There were two primary aims of this study. The first aim was to investigate the reciprocal relationship between PIU [including two specific forms: PSMU and problematic gaming (PG)] and psychological distress among schoolteachers using a cross-lagged panel model. The selection of PSMU and PG as PIU variables was based on the fact that these are relatively common types of PIU (36, 37) and there are already well-developed psychometric instruments to assess these behaviors (38). The second aim was to examine the associations between school administrators' support, schoolteachers' PNT of online teaching, psychological distress, and PIU using Hierarchical Linear Modeling (HLM). Here, administrators' support was at the school level, and schoolteachers' mental health (including PNT of online teaching and psychological distress) and PIU were at the individual (teacher) level. The conceptual framework and research hypotheses are described in the following section.

## Conceptual framework

**Figure 1:**
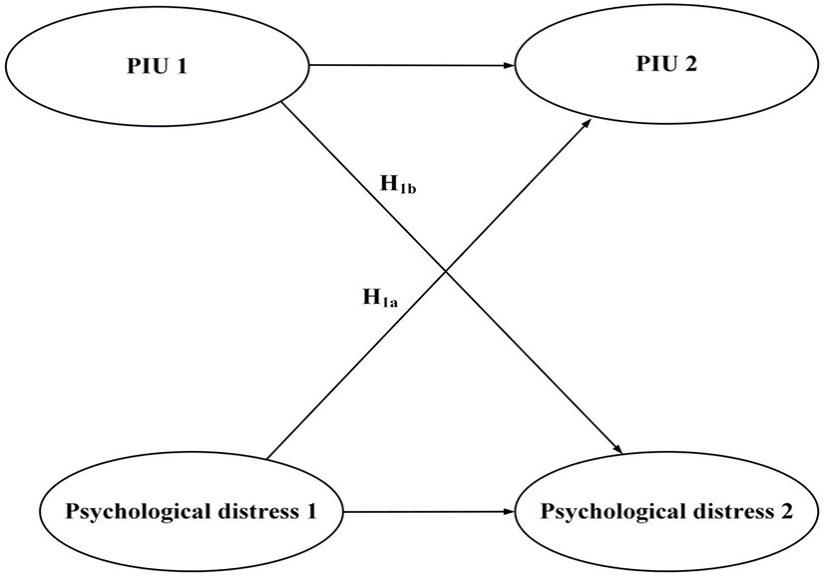
Framework for the reciprocal relationship between PIU and psychological distress; variables include psychological distress and two forms of problematic internet use (PIU): problematic social media use and problematic gaming.

**Figure 2:**
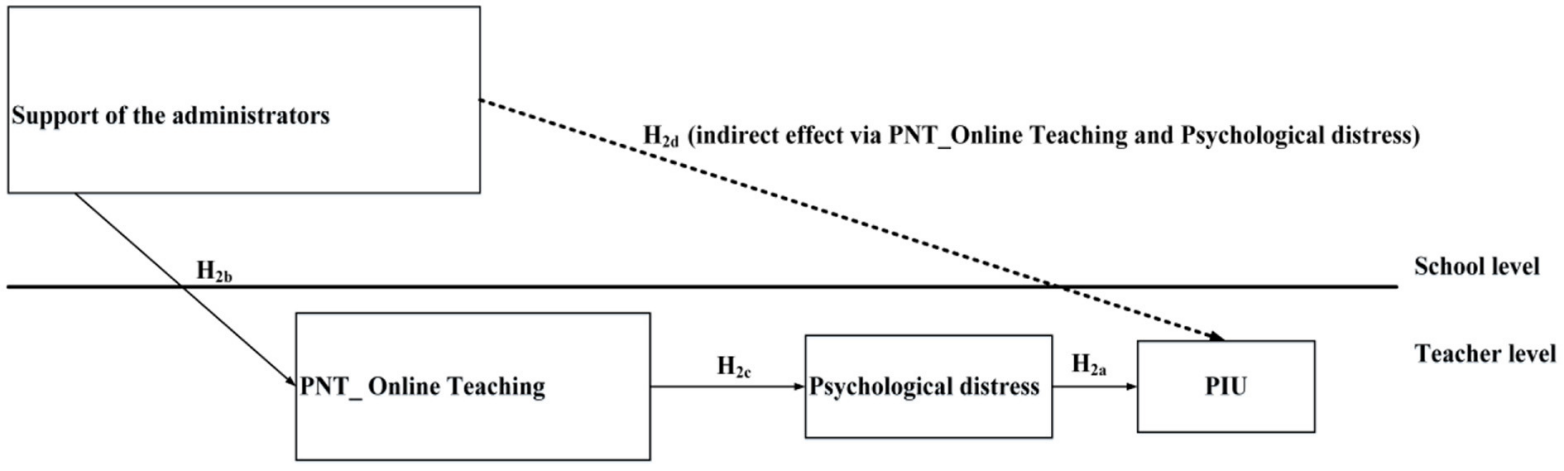
Framework of the cross-level serial mediation model; all variables were assessed at Time 1, including administrator support, psychological need thwarting of online teaching (PNT_Online Teaching), psychological distress, and two forms of problematic internet use (PIU): problematic social media use and problematic gaming.

Our research framework includes two conceptual models (see Figures 1, 2). The first model involves the reciprocal relationship between PIU and psychological distress. In this model, longitudinal data was used to verify our research hypotheses. The second model involves cross-level mediation (including the variables of administrators' support, PNT of online teaching, psychological distress, and PIU) and was tested using cross-sectional data.

### The reciprocal relationship between PIU and psychological distress

In regard to the reciprocal relationship between PIU and psychological distress, the Interaction of Person-Affect-Cognition-Execution (I-PACE) model proposed by Brand et al. (39, 40) was used to explain the mechanism in which the psychological distress of teachers in stressful environments leads to the development of PIU. Subsequently, a bidirectional lens (18) was selected to interpret the potential reciprocal relationship between PIU and psychological distress, wherein PIU leads to psychological distress.

The I-PACE model is a framework to explain how PIU activities develop (39, 40) and has received empirical support (41, 42). More specifically, the I-PACE model proposes the impact of major influencing variables on PIU, including predisposing background variables [i.e., underlying individual differences, such as personality, specific motives for using the excessive behaviors (e.g., use of the internet or smartphones for stress relief)], affective and cognitive responses, and executive functioning impairment. According to the I-PACE model, under stressful environments, some individuals will experience an emotional response, such as psychological distress. Consequently, these emotional responses can lead to addictive behaviors in some individuals, such as PSMU and PG (explained in the I-PACE model as behaviors caused by a reduction of executive functioning and loss of self-control). In the I-PACE model, increased psychological distress reduces some individuals' ability to inhibit their cravings for (and addiction to) social media use and online gaming. Finally, given diminished inhibitory control, some individuals increase their time spent on social media use and online gaming, which can become problematic in nature.

In the context of school closure, many schoolteachers were motivated to using Wi-Fi-enabled digital devices to help cope with stressors resulting from the pandemic (9, 41, 43). Moreover, with increased work hours (resulting from mandatory online teaching tasks with increased burdens and challenges) and other issues (e.g., fear of COVID-19, home restrictions, lack of support for conducting online teaching, or the thwarting of psychological needs due to online teaching), mandatory online teaching tasks created a stressful environment (9–13, 44) which resulted in psychological distress among many teachers, as a type of emotional response. According to the I-PACE model, increased psychological distress is likely to reduce teachers' inhibitory control over problematic online behaviors.

Moreover, in addition to the influence of psychological distress on individuals' PIU, PIU might also inversely increase psychological distress. This bi-directional association has been confirmed in recent studies (19, 45). As addictive behaviors develop, PIU may inversely enhance an individual's negative emotions as a result of their inability to adapt to their surroundings. This can lead to persistent negative effects on their mental health (46, 47). This is especially true for schoolteachers as recent findings showed that teachers' workloads were still heavy in the initial period after school campuses reopened (4, 6, 48). Addictive behaviors, potentially a response to negative emotions during school closure periods, may inhibit teachers' adaptation when facing normal offline teaching duties and may lead to increased psychological distress. Based on this literature, we hypothesized that there would be a reciprocal relationship between PIU and psychological distress. More specifically, the hypotheses (H_s_) were: (i) teachers' initial psychological distress would demonstrate a positive association with subsequent PIU (H_1a_), and (ii) teachers' initial PIU would demonstrate a positive association with subsequent psychological distress (H_1b_).

### The cross-level mediation model

In relation to the second aim (i.e., to examine the associations between school administrators' support, schoolteachers' PNT of online teaching, psychological distress, and PIU), cross-level serial mediation was hypothesized, such that school administrators' support served as the independent variable with schoolteachers' PNT of online teaching and psychological distress as mediators (PNT of online teaching was the first mediator and psychological distress was the second mediator, affected by PNT of online teaching) with the two forms of schoolteachers' PIU as dependent variables. The model is illustrated as Figure 2. Among the paths in the model, according to I-PACE model explained above, a positive association between psychological distress and teachers' PIU was hypothesized (H_2a_). The following paragraphs provide an overview of the other paths in this serial mediation model.

Before introducing the paths included in the cross-level serial mediation model, a brief justification is required as to why PNT was hypothesized as resulting from online teaching during school closure. In numerous surveys, teachers have expressed frustration of their psychological needs as a result of mandatory online teaching, although these studies did not exclusively use the term “PNT”. For example, Yi et al. (9) and Collie (35), who used the theory of PNT as their framework, and some qualitative findings (i.e., interviews with teachers) have demonstrated high levels of frustration related to the competence of using technology for online teaching (11, 16). Moreover, teachers were seldom included in the decision-making process on how to conduct online teaching (27). In essence, such findings relate to PNT. Furthermore, in a recent study, Kulikowski et al. (28) used the title “E-learning? Never again!” to describe the context in which teachers were forced to teach online and the possibility of unintended consequences arising from this situation. In that study, core elements of PNT, such as decreased perceived competence, limited autonomy, and restricted social interaction (relatedness) were all reported as reasons for low work motivation among teachers. Consequently, based on the aforementioned literature, in the present study we presumed that PNT was a substantial during online teaching.

Regarding the paths included in the cross-level serial mediation model, given that the thwarting of psychological needs is affected by the surrounding environment (29, 48), it is natural to consider that the support of school administrators may help alleviate teachers' PNT during online teaching. In the literature, when employees feel that their organizations treat them simply as tools, this organizational dehumanization contributes to employees' PNT and, indirectly, increases in psychological strain (49). Pressure from school administrators (including time constraints and performance evaluation) can result in the thwarting of teachers' psychological needs (50). Based on the aforementioned literature, we hypothesized that greater support from administrators would be associated with lower levels of teachers' PNT related to online teaching (H_2b_).

Regarding the influence of PNT from online teaching on teachers' psychological distress, according to Self-Determination Theory, PNT is a risk factor for poor psychological wellbeing (29–31), with several empirical studies reporting adverse effects on teachers' mental health as a result of PNT. For example, PNT was significantly associated with burnout among Spanish schoolteachers (30). Moreover, in the context of the COVID-19 pandemic, Yi et al. (9) found that Chinese teachers' PNT from online teaching was positively associated with psychological distress. In a study conducted with Australian teachers, leadership types that frustrated teachers' autonomy (i.e., autonomy-thwarting leadership) increased teachers' emotional exhaustion (35). Consequently, we hypothesized that higher levels of PNT from online teaching would result in an increase in teachers' psychological distress (H_2c_).

Finally, considering the aforementioned research hypotheses (H_2a_, H_2b_, and H_2c_), we expected that increased administrative support would first alleviate teachers' psychological needs thwarting of online teaching which would then lower psychological distress. Through serial mediation, higher levels of administrative support would decrease teachers' PIU. Consequently, we hypothesized that administrative support would have a negative indirect effect on teachers' PIU through the serial mediators (i.e., PNT of online teaching and psychological distress) (H_2d_).

## Methods

### Participants

The present study was conducted in a city in the Jiangxi Province of China and was approved by the Jiangxi Psychological Consultant Association (IRB ref: JXSXL-2020-J013). Contrary to the relatively lax restrictions concerning COVID-19 in many other countries, as of the time of writing, the Chinese government is still adopting a zero-COVID-19 policy to prevent the spread of COVID-19 infections. For example, if a COVID-19 infected individual is identified in a city, the local government tends to take a strict approach and closes public places and schools. At the end of October 2021, several COVID-19 infections were identified in the aforementioned city in Jiangxi Province and the city government decided to fully implement online teaching in primary and secondary schools across the whole city, and not allow any face-to-face physical courses (starting November 3, 2021). In this sudden move to online teaching, primary and secondary schools were required to offer all courses on specified online learning platforms. The implementation of online courses in primary and secondary schools differed. Online courses in secondary schools were conducted through live classes. In primary schools, based on the characteristics of different subjects, online courses were offered through various teaching methods. For example, in Chinese language classes, students were asked to watch pre-recorded videos explaining the content of their textbook. In physical education classes, children were asked to record teacher-specified actions and upload these video files to an online learning platform. Some courses (such as mathematics) were offered in the form of a combination of live lectures by teachers and asynchronous student exercises. In co-operation with the city's education authority, two weeks after the full implementation of online teaching, an online survey adopting purposive sampling was administered to assess schoolteachers' mental health status during this quarantine period (Time 1: mid-November, 2021). Subsequently, after a month of rigid restrictions, the pandemic outbreak was brought under control in the city and campuses reopened in mid-December, with teachers returning to in-person teaching. A follow-up online survey was conducted in early-January, 2022 (Time 2), ~2 weeks after the campuses reopened.

The data for the online survey were collected via a hyperlink which was sent to primary and secondary schoolteachers with the assistance of local education authorities. The survey was voluntary and asked participants to leave their email address if they would like to participate in the follow-up survey. Electronic informed consent was obtained on the first page of the online survey. A total of 1,642 school teachers provided their email addresses and participated in the longitudinal study. Given that HLM analysis required a grouping variable (ID) for evaluating and linking nested data (i.e., teachers' affiliation with a specific school), we removed responses from the participants who did not clearly disclose their school's name (e.g., the school's name was not written, was not legible, or was misspelled). The final sample for statistical analysis comprised 980 primary and secondary schoolteachers from 115 schools. Apart from the question asking for the school's name, participants were required to complete all other questions on each page of the survey in order to move on to the next page. As such, there were no missing data. Furthermore, we also confirmed that sample attrition was not systematic and would not have an obvious effect on the results (a detailed examination is provided in Supplementary Table S1 with note).

### Measures

In the present study, demographic variables [i.e., age, sex, and school type (primary or secondary school)] were collected as the control variables for the cross-lagged panel model and HLM analysis. Regarding other measures, administrators' support and PNT of online teaching, participants were asked to answer questions in relation to ongoing mandatory online teaching at that time. For psychological distress and the two specific forms of PIU, participants were asked to answer questions considering their condition over the past month. Among these measures, except for administrators' support and PNT of online teaching (which were assessed at Time 1), other variables were assessed at both Time 1 and Time 2. The instruments used are described in the following subsections.

#### Administrators' support

Because there is no instrument that exclusively evaluates support from school administrators in a mandatory online teaching setting, we referred to the Scale of Technology Users' Beliefs (STUB) developed by Nistor et al. (51) to develop a new instrument for this study. More specifically, the STUB assessed how important individuals (e.g., line managers, colleagues, and friends) would expect them to use technology (e.g., “*People who are important to me think that I should use the computer as a learning tool”; “The senior management has been helpful in the use of the computer as a learning tool”*). In terms of the principles of item development for the present study, the wording of the original STUB was retained for items assessing positive attitudes of important individuals toward technology use and the provision of help and resources for people using technology. In addition, the context of the items was revised to reflect the online teaching environment during the pandemic. Four items were then developed for the present study and were rated on a five-point Likert-type scale from 1 (*strongly disagree*) to 5 (*strongly agree*). The four items were: “*Administrators want teachers to be able to teach online smoothly during the outbreak”, “School administrators provided most of the necessary resources to help teachers be able to teach online during the outbreak”, “Administrators always support and encourage teachers to use online teaching during the pandemic”*, and “*Administrators understand the benefits of using online teaching during the pandemic”*. The internal reliability was very good in this present study (Cronbach's α = 0.85, McDonald's ω = 0.85). The uni-dimensional structure of the adapted Administrators' Support Scale was validated using confirmatory factor analysis (CFA) with the estimation of diagonally weighted least squares (DWLS) since DWLS has less bias and more accurate performance than maximum likelihood (ML) and Robust ML in CFA when the observed indicators are ordinal (e.g., the Likert-type scale used in the present study) (52). The results of CFA showed that factor loadings that were all higher than 0.56 and the overall model had a good fit with the data [i.e., Comparative fit index (CFI), non-normed fit index (NNFI), root mean square error of approximation (RMSEA), and standardized root mean square residual (SRMR) were 0.99, 0.98, 0.05, and 0.04 respectively]. Furthermore, because the aggregated value of schoolteachers' perceived support from the administrators was used to represent this construct at school-level, the within-group agreement (*r*_wg_) was justified and acceptable agreement was obtained in terms of the score on the four items (*r*_wg_ = 0.88). Consequently, it was deemed reasonable to aggregate the values from teacher-level responses to reflect a school-level factor.

#### Psychological need thwarting of online teaching

The Psychological Need Thwarting Scale of Online Teaching (PNTSOT) was used to assess schoolteachers' psychological need thwarting toward online teaching during the period of school closure (Time 1). The PNTSOT was specifically designed to assess the extent of psychological need thwarting during online teaching tasks (9). The PNTSOT comprises three subscales (i.e., autonomy, competence, and relatedness thwarting). Example items include: “*I have to follow a prescribed online teaching style (way) during the pandemic”* (autonomy thwarting), “*Online teaching during the pandemic sometimes makes me feel powerless”* (competence thwarting) and “*I feel disconnected from other colleagues and leaders when teaching online during the pandemic*” (relatedness thwarting). Items are rated on a seven-point Likert-type scale from 1 (*strongly disagree*) to 7 (*strongly agree*), and higher scores indicates more serious psychological need thwarting during online teaching tasks. A sound factorial validity was found in Yi et al.'s (9) study among schoolteachers (CFI = 0.97, NNFI = 0.95, RMSEA = 0.09, and SRMR = 0.05). The internal reliability of the PNTSOT was very good in the present study (Cronbach's α = 0.88, McDonald's ω = 0.88). Following other studies (9, 49), the three kinds of PNT were treated as an overall construct, therefore individual subscales of PNT were not included in the data analysis.

#### Psychological distress

The Depression, Anxiety, and Stress Scale (DASS-21) developed by Lovibond and Lovibond (53) was used to assess schoolteachers' psychological distress at both Time 1 and Time 2, given that recent studies have demonstrated the overall score of DASS-21 reflects general psychological distress rather than distinct emotional disorders (54, 55). Items in the DASS-21 are rated on a four-point scale from 0 (*never*) to 3 (*almost always*) with higher scores indicating higher levels of psychological distress. The summed score of all items in the DASS-21 was used for further processing during HLM analysis. The Chinese version of the DASS-21 has satisfactory psychometric properties (56, 57). In this present study, the internal reliability of the DASS-21 was excellent (Cronbach's α = 0.94, McDonald's ω = 0.95 at Time 1 and Cronbach's α = 0.96, McDonald's ω = 0.96 at Time 2).

#### Problematic social media use

The Bergen Social Media Addiction Scale (BSMAS) was used to assess schoolteachers' PSMU. The BSMAS was developed by Andreassen et al. (58) and comprises six items (e.g., “*I feel an urge to use social media more and more”*) rated on a five-point Likert-type scale from 1 (*very rarely*) to 5 (*almost always*). Higher scores indicate greater risk of problematic social media use. The Chinese BSMAS has demonstrated very good psychometric properties (59, 60). The internal reliability of the BSMAS in the present study was satisfactory (Cronbach's α = 0.86, McDonald's ω = 0.87 at Time 1 and Cronbach's α = 0.88, McDonald's ω = 0.88 at Time 2).

#### Problematic gaming

The nine-item Internet Gaming Disorder Scale (IGDS-SF9) was used to assess the level of PG among schoolteachers. The IGDS-SF9 was developed by Pontes and Griffiths (46) and has a unidimensional structure. Items (e.g., “*Do you feel the need to spend increasing amount of time engaged gaming in order to achieve satisfaction or pleasure?*”) on the IGDS-SF9 are rated on a five-point Likert-type scale from 1 (*strongly disagree*) to 5 (*strongly agree*) with higher scores indicating more problematic gaming. The Chinese IGDS-SF9 has good psychometric properties (59, 60). In this present study, the internal reliability of IGDS-SF9 was excellent (Cronbach's α = 0.95, McDonald's ω = 0.95 at Time 1 and Cronbach's α = 0.96, McDonald's ω = 0.96 at Time 2).

### Data analysis strategy

Descriptive statistics and Pearson correlations were first used to analyze the characteristics of the participants and the association among all study variables, respectively. For H_1a_ and H_1b_, a cross-lagged panel model with LISREL 8.80 on PSMU and PG was conducted. The analysis first evaluated the overall model fit indices and then scrutinized the significance of the path coefficients reflecting the cross-lagged effects. The criteria for evaluating model fit included a comparative fit index (CFI), a non-normal fit index (NNFI), a root mean square error of approximation (RMSEA), and a standardized root mean square residual (SRMR). Model fit was considered acceptable when the following criteria were met: RMSEA values of 0.08 or lower, SRMR values of 0.08 or lower, CFI values of 0.95 or higher, and NNFI values of 0.95 or higher (61). In terms of H_2a_, H_2b_, and H_2c_, a cross-level serial mediation model with HLM 7.0 was conducted on PSMU and PG, respectively. Before testing H_2a_, H_2b_, and H_2c_, a fully unconditional model (null model, without adding any explanatory variables) was used to calculate the intra-class correlation (ICC). If the ICC exceeded 0.059, HLM analysis was considered appropriate (62). Subsequently, for testing H_2a_, H_2b_, and H_2c_, HLM equations for the three path coefficients are shown below.

For H_2a_: Teacher-level: PIU _ij_ = β_0j_ + β_1j_(sex _ij_) + β_2j_(age _ij_) + β_3j_(psychological distress _ij_) + γ_ij_

School-level: β_0j_ = γ_00_ + γ_01_ (school type _j_) + U_0j_, β_1_ = γ_10_, β_2j_ = γ_20_, β_3j_ = γ_30_

For H_2b_: Teacher-level: PNT of online teaching _ij_ = β_0j_ + β_1j_(sex _ij_) + β_2j_(age _ij_) + γ_ij_

School-level: β_0j_ = γ_00_ + γ_01_ (school type _j_) + γ_02_(administrator support _j_) + U_0j_, β_1_ = γ_10_, β_2j_ = γ_20_

For H_2c_: Teacher-level: Psychological distress _ij_ = β_0j_ + β_1j_(sex _ij_) + β_2j_(age _ij_) + β_4j_(PNT of online teaching _ij_) + γ_ij_

School-level: β_0j_ = γ_00_ + γ_01_ (school type _j_) + U_0j_; β_1_ = γ_10_, β_2j_ = γ_20_, β_4j_ = γ_40_.

Finally, in order to test H_2d_, the bootstrap method (63) with 5,000 random samples was used to test the indirect effect from administrator support on two forms of PIU through the serial mediators (PNT of online teaching and psychological distress). More specifically, the path of interest is labeled as 2-1-1-1 model since the antecedent variable (independent variable; administrator support) was assessed at level-2 (school level), while the two mediators and dependent variables were assessed at level-1 (individual teacher level; PNT of online teaching, psychological distress, PIU). According to Zhang et al., for 2-1-1-1 models, where the cross-level mediation of interest could exist only across level-2 units, they recommend that the estimation of the indirect effect should be conducted separately for between-group and within-group effects, rather than combining them into a single estimate (64). Following Zhang et al.'s suggestion, the test of the serial mediation effect was conducted at the school level. Namely, aggregated PNT of online teaching, and aggregated psychological distress mediates the relationship between administrators' support and aggregated PIU. Model 6 in Hayes' PROCESS macro (65) was used to test the significance of the indirect effect. The bootstrapping procedure was used in the PROCESS macro to estimate the path parameters and the confidence interval (CI) of the indirect effect in this serial mediation model. We used bootstrapping with 95% bias-corrected and accelerated (BCa) confidence intervals for 5,000 random samples.

Relevant assumptions for statistical analysis, including linearity, normality, and homoscedasticity of residuals, were confirmed with a scatter plots (for linearity and homoscedasticity of residuals) and Quantile-Quantile (Q-Q) plots (for normality of residuals). The residual scatter plots (residuals vs. predicted; see Supplementary Figures S1, S2) and Q–Q plots (see Supplementary Figures S3, S4) showed that there was no obvious non-linear pattern for the residual plots, and an approximately normal distribution of residuals was found. However, homogeneity of variance was violated according to the pattern illustrated in Supplementary Figures S1, S2. To provide unbiased estimation for the coefficients, results with robust standard errors (SE) displayed in HLM 7.0 are reported. Results from HLM 7.0 produce two tables containing the coefficient parameters for ordinary SEs and robust SEs. In cases where data distributions violate the assumption of homogeneity of variance, it is recommended that the results are reported for robust SEs (66).

## Results

### Descriptive statistics and Pearson correlations

Table 1 presents the demographic characteristics of the participants in the present study. The average age of the participants was 34.76 years, mostly teaching in primary schools (76.8%), with the majority being female (82.9%). In terms of years of working experience, teachers with <10 years of experience (47.3%) and more than 10 years of experience (52.7%) were relatively balanced. The demographic variables in the present sample were close to the overall population statistics (i.e., from all primary and secondary schoolteachers in mainland China) (67) in relation to age (mean age for the population of schoolteachers is 37.78), school type (64% of schools nationwide are primary schools), and gender (70% of schoolteachers are female).

**Table 1:** Characteristics of participants.

	***N* = 980**
Age in years; M (SD)	34.76 (8.22)
**School type;** ***n*** **(%)**	
Primary school	753 (76.8%)
Secondary school	227 (23.2%)
**Gender;** ***n*** **(%)**	
Male	168 (17.1%)
Female	812 (82.9%)
**Years of teaching experience;** ***n*** **(%)**	
Under 5 years	222 (22.7%)
6–10 years	241 (24.6%)
11–15 years	188 (19.1%)
16–20 years	108 (11.0%)
Over 20 years	221 (22.6%)
**Teaching subject;** ***n*** **(%)**	
Chinese	387 (39.5%)
English	108 (11.0%)
Mathematics	333 (34.0%)
Science	36 (3.6%)
Social science	32 (3.2%)
Other (e.g., music, art, physics, politics)	84 (8.7%)

Table 2 displays the means (and *SDs*) and Pearson correlation coefficients of the variables in this study. The results of the Pearson correlations showed that, except for the association between administrators' support and PSMU at Time 1 being statistically nonsignificant, all other paired relationships were statistically significant. Among these coefficients, negative relationships were found for administrators' support and PNT of online teaching, psychological distress, and PIU (*r* = −0.30 to −0.08). PNT of online teaching, psychological distress, and PIU were all mutually and positively significant (*r* = 0.18–0.44).

**Table 2:** Descriptive statistics and Pearson correlation matrix of the study variables.

	**Mean (SD)**	**Cronbach's α**	**McDonald's ω**	**1**	**2**	**3**	**4**	**5**	**6**	**7**	**8**
1. Administrators' support	15.33 (2.68)	0.85	0.85	1.00							
2. PNT of online teaching^a^	40.31 (11.35)	0.88	0.88	−0.30^**^	1						
3. Psychological distress_Time 1^b^	18.94 (18.92)	0.94	0.95	−0.17^**^	0.35^**^	1					
4. Psychological distress_Time 2^b^	18.72 (21.69)	0.96	0.96	−0.15^**^	0.25^**^	0.61^**^	1				
5. PSMU_Time 1^c^	14.09 (4.74)	0.86	0.87	−0.01	0.22^**^	0.32^**^	0.21^**^	1			
6. PSMU_Time 2^c^	13.35 (4.55)	0.88	0.88	−0.08^*^	0.19^**^	0.28^**^	0.35^**^	0.44^**^	1		
7. PG_Time 1^d^	12.92 (5.49)	0.95	0.95	−0.19^**^	0.18^**^	0.36^**^	0.26^**^	0.27^**^	0.24^**^	1	
8. PG_Time 2^d^	13.59 (6.22)	0.96	0.96	−0.19^**^	0.21^**^	0.32^**^	0.43^**^	0.22^**^	0.44^**^	0.50^**^	1

* *p* < 0.05,

** *p* < 0.01.

a PNT of online teaching = Psychological need thwarting of online teaching, assessed using Psychological Need Thwarting Scale of Online Teaching (PNTSOT).

b Assessed using Depression, Anxiety, Stress Scale-21 multiplied by 2.

c PSMU = Problematic social media use, assessed using Bergen Social Media Addiction Scale (BSMAS).

d PG = Problematic gaming, assessed using Internet Gaming Disorder Scale-Short Form (IGDS-SF9).

### Cross-lagged panel model

**Figure 3:**
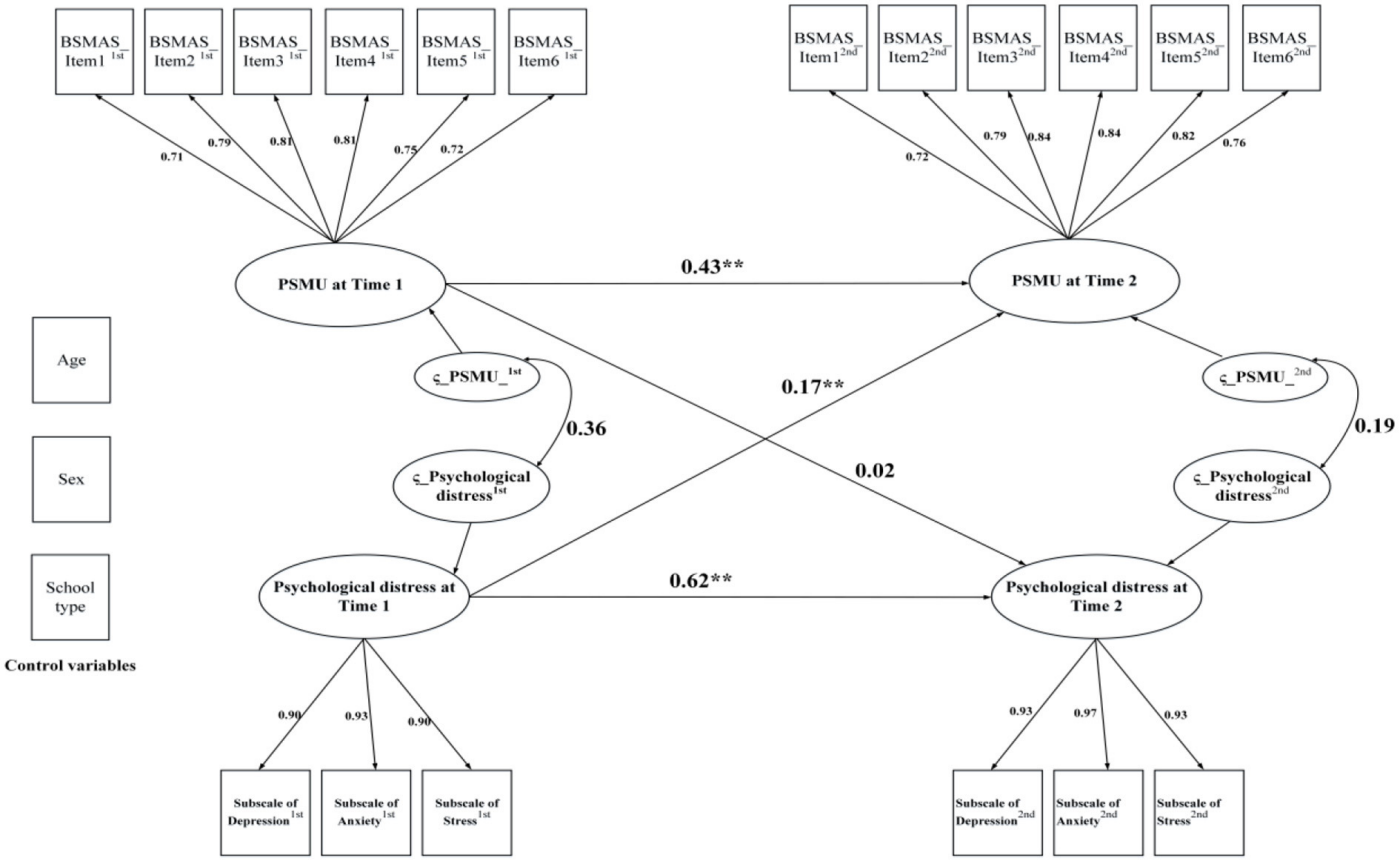
Cross-lagged panel model of problematic social media use and psychological distress; PSMU, Problematic social media use; BSMAS, Bergen Social Media Addiction Scale. **p < 0.01.

**Figure 4:**
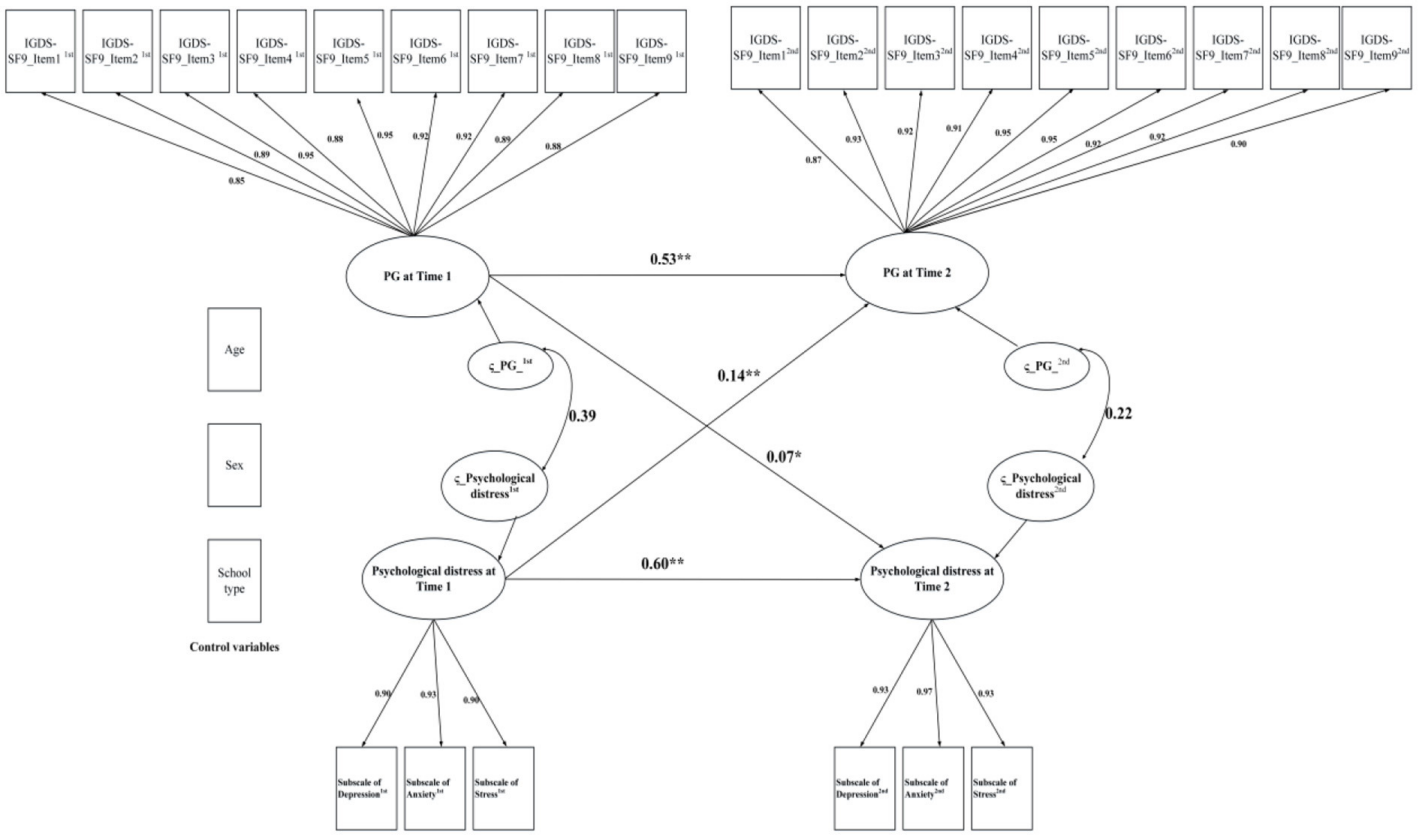
Cross-lagged panel model of problematic gaming and psychological distress; PG, Problematic gaming; IGDS-SF9, Nine-item Internet Gaming Disorder Scale; *p < 0.05, **p < 0.01.

Regarding the results of cross-lagged panel models including control variables (i.e., age, sex, and school type), the model for PSMU and PG had acceptable model fit indices except for RMSEA, which was slightly higher than the criterion (0.08): PSMU: χ^2^ (162) = 1,287.85, CFI = 0.959, NNFI = 0.947, RMSEA = 0.083, and SRMR = 0.052; PG: χ^2^ (292) = 2,495.28, CFI = 0.973, NNFI = 0.968, RMSEA = 0.087, and SRMR = 0.027. Subsequently, data from the cross-lagged panel model (see Figures 3, 4) found that psychological distress was significantly associated with both PSMU (β = 0.17, *p* < 0.01) and PG (β = 0.14, *p* < 0.01), which supported H_1a_. However, since cross-lagged panel model analysis found that PG had a significant effect on psychological distress (β = 0.07, *p* = 0.03) whereas PSMU did not (β = 0.01, *p* = 0.28), the data only partially supported H_1b_.

### The cross-level mediation model

For the cross-level mediation model (see Table 3), ICCs were 0.0537, 0.0709, 0.0055, and 0.0046, respectively, for the null models which treated PNT of online teaching, psychological distress, PSMU and PG as outcomes. The results showed that considerable ICCs were found for PNT of online teaching and psychological distress, which were close to or exceeding 0.059, whereas small ICCs were found for the two types of PIU. Despite a minimal clustering effect for PIU, it is still necessary to consider the intra-group effect for teachers' PNT of online teaching and psychological distress. Moreover, studies have also cautioned that small ICCs may not warrant abandoning HLM, since additional dependence on higher-level groupings can arise after explanatory variables are entered into the models (68, 69). As such, considering that small values for ICCs should not immediately rule out the use of multi-level analysis (70), HLM was considered suitable for use in addressing this research aim.

**Table 3:** Unstandardized coefficients (SE) obtained from hierarchical linear modeling using robust SEs.

**Parameter**	**Model for ICC_PNT_ online teaching**	**Model for ICC_PD**	**Model for ICC_PSMU**	**Model for ICC_PG**	**Model for H_2a__PSMU**	**Model for H_2a__PG**	**Model for H_2b_**	**Model for H_2c_**
**Fixed effects**
Intercept γ_00_	40.33 (0.45)^**^	9.49 (0.31)^**^	14.09 (0.15)^**^	12.92 (0.16)^**^	15.18 (0.97)^**^	16.02 (1.00)^**^	44.23(2.16)^**^	10.29(2.15)^**^
γ_01_ School type (secondary school)					−0.16 (0.39)	0.02 (0.34)	−1.21(1.02)	0.17(0.74)
γ_02_ Support							−2.18(0.34)^**^	
γ_10_ Sex (male)					−0.44 (0.41)	−1.73 (0.49)^**^	−1.06(0.97)	−0.62(0.98)
γ_20_ Age					−0.06 (0.02)^**^	−0.07 (0.02)^**^	0.03(0.05)	−0.003(0.04)
γ_30_ PD					0.15 (0.01)^**^	(0.20 0.02)^**^		
γ_40_ PNT_ Online Teaching								0.29(0.03)^**^
Random effects
σ^2^ (within-group variation)	114.42	88.90	16.38	17.14	12.63	12.43	100.61	76.89
τ_00_ (between-group variation)	6.49	6.78	0.09	0.08	0.16	0.23	9.72	10.60
ICC	0.0537	0.0709	0.0055	0.0046				

ICC, Intra-class correlation; PD, Psychological distress; PNT_Online Teaching, Psychological need thwarting of online teaching; PSMU, Problematic social media use; PG, Problematic gaming.

** *p* < 0.01.

Regarding the results of HLM, there was a positive association between teachers' psychological distress and the two types of PIU (PSMU: *B* = 0.15, SE = 0.01, *p* < 0.01; PG: *B* = 0.20, SE = 0.02, *p* < 0.01), providing support for H_2a._ Furthermore, the results showed a significant and negative effect of administrator support on teachers' PNT of online teaching (*B* = −2.18, SE = 0.34, *p* < 0.01), providing support for H_2b_. Moreover, HLM analysis also showed a significant and positive effect for PNT of online teaching on psychological distress (*B* = 0.29, SE = 0.03, *p* < 0.01), supporting H_2c_. For testing H_2d_, using bootstrapping with 5,000 random samples, the results demonstrated that the serial indirect effect was significant [indirect effect = −0.12, 95% CI (−0.18, −0.07)] (administrators' support → PNT of online teaching → psychological distress → PG), whereas other indirect effects of administrators' support on PG with PNT of online teaching or psychological distress as sole mediators were not significant. Finally, no serial mediation effect was found for administrators' support on PSMU. Likewise, no other indirect effects via solo mediators were found for PSMU. Consequently, the data only partially supported H_2d_.

## Discussion

While online learning is an emergency response to sustain learning, its impact on faculty and students is also of considerable concern. More specifically, in the early months of the global outbreak (i.e., March to June 2020), many studies described various situations encountered by teachers when rushing to teach online, including insufficient digital resources (e.g., no available e-books) (12, 16, 27, 71), inadequate infrastructure (internet access) (16, 72), increased work time due to the technology restrictions (16, 72), inexperience with integrating technology into teaching (11, 73, 74), insufficient autonomy of online teaching (27, 28), redundant work and high overlap of work and personal time (75–77), and anxiety about the lives and learning of specific disadvantaged (low socioeconomic status) students (73, 75, 78). These phenomena, combined with general fear of the pandemic, restrictions on physical activity imposed by home quarantine measures, and possible excessive internet use (24, 25), could easily lead to a negative impact on teachers' mental health. Therefore, the present study used a longitudinal study design with a relatively large sample size to investigate mental health issues among schoolteachers during the COVID-19 pandemic. The findings demonstrated that (i) schoolteachers' psychological distress and PG had a reciprocal relationship, although this bidirectional association was not significant for PSMU as a predictor of psychological distress, despite the fact that psychological distress did predict PSMU; and (ii) serial mediation provided support for the potential influence of administrators' support on schoolteachers' PG via the mediators of PNT of online teaching and psychological distress. Interestingly, there was no significant indirect effect of administrator support through serial mediation on PSMU. In fact, according to the correlation matrix in Table 2, PSMU was the only variable not significantly correlated with administrator support. This finding, along with the lack of an effect of PSMU at Time 1 on psychological distress at Time 2, deserves further evaluation.

Concerning hypotheses tested in the cross-lagged panel model, most schools around the world adopted mandatory online teaching to maintain uninterrupted learning (1, 2, 5, 79) which exposed schoolteachers to high levels of stress and may have induced inappropriate coping methods among some individuals (e.g., PIU). The findings here lend support to the I-PACE model (39, 40) and the bidirectional lens adopted in prior studies (18, 19, 45). For example, prior studies have reported increased PIU among normal adults during home restriction (80–82) and the COVID-19 literature also shows that for individuals who needed to work from home, PIU was significantly correlated with psychological distress during this period (83, 84). However, most research assessing the associations between psychological distress and PIU has focused on children (85–88), adolescents (89, 90), and individuals with existing psychiatric illnesses (91, 92). Rarely has such evidence been reported among individuals with specific occupations, such as schoolteachers. Therefore, the present study's findings extend the current literature in an understudied, and potentially vulnerable, population (i.e., schoolteachers). Notably, despite the fact that the adverse effect of PG on teachers' psychological distress was relatively small, the significant association reported in our findings is sufficient to suggest that it is necessary to be aware of schoolteachers' PIU and provide guidance to avoid PIU in addition to assisting teachers in managing psychological distress across different pandemic periods. In fact, the relatively lower association between PIU and psychological distress may be due to the age of the participants in the sample, as Lathabhavan and Padhy found that adults over the age of 40 (Generation X, similar to our participants) had significantly lower PIU-stress associations than those aged 10–35 (Generations Y and Z) (84).

A notable finding of this study was that the PG assessed at the time of school closure had a greater lasting negative impact on teachers' mental health after returning to campus as compared with PSMU. Internet gaming and social media use have different characteristics. We speculate that, for Chinese teachers, the use of social media often involves dealing with day-to-day work tasks (such as interacting with parents in online communities and dealing with work tasks assigned by leaders in online teacher work groups), and such online behavior would not unduly hinder teachers' work adaptation after returning to offline teaching. Comparatively, internet gaming is mainly a competitive game activity, in which teachers, as players, often need to spend a lot of time and energy in this virtual world in order to win, which is more likely to hinder their adaptation when returning to an offline work environment. Consequently, higher severity psychological distress was induced by PG. Our finding is in line with Wang et al. (93) and Pontes (94) in which PG contributes more harm to mental health than PSMU. Despite this, the effects of these two forms of PIU on individual mental health require further investigation, as some studies have yielded inconsistent results [e.g., no clear pattern between PG and PSMU in terms of clinically relevant depressive symptoms in Wartberg et al. (95)].

Given that the hypotheses regarding the cross-level mediation model were partial supported, this confirms the importance of administrative support on teacher wellbeing as detailed in many past studies (1, 5, 10, 16, 75) which could indirectly reduce the extent of PIU (especially PG) among schoolteachers. There are several reasons for this, including organizational dehumanization, the pressure of school authority, and the anxiety of communicating with administrators that produce an increased level of PNT and daily anxiety among employees (10, 49, 50). Moreover, supportive leadership has been associated with greater work buoyancy and reduced stress among schoolteachers (35). Consequently, the present study speculates that administrators' support would first alleviate the PNT of online teaching, consequently lowering teachers' psychological distress. As such, with lower frustration of psychological needs and increased mental health, lower levels of PG would be reported among schoolteachers. In order to find an indirect effect of administrators' support on PG, serial mediators were necessary. In fact, if sole mediators were included (either PNT of online teaching or psychological distress), this indirect effect was not significant. This finding highlights the unique contribution of this study wherein serial mediation demonstrates how administrators' support alleviated teachers' PG as assessed during the onset of the pandemic. However, since the direct relationship between administrator support and psychological distress was not tested, only partial mediation can be stated with a degree of certainty. The development of alternative models wherein administrator support is directly associated with other variables of interest is recommended for future research in this area.

As noted above, there are some limitations in the present study which must be addressed. First, because the sample was not drawn randomly, it is possible that it does not represent the entire population of primary and secondary schoolteachers in mainland China. Moreover, the participants in our study were all primary or secondary schoolteachers. Therefore, the present findings cannot be generalized to teachers working in other schools (e.g., kindergartens or colleges). Second, the present sample was recruited from mainland China, which adopted different policies in COVID-19 pandemic control as compared to other countries (e.g., China's zero-COVID-19 policy). Therefore, the impacts of the COVID-19 pandemic on the variables found in the present study (i.e., psychological distress and PIU) might be different in other contexts. Therefore, studies using samples from other countries are needed to corroborate the findings reported here. Third, all the variables in this study were collected using self-report data, introducing potential biases such as memory recall and social desirability, which are difficult to avoid. Therefore, more objective measures for PIU, such as the total amount of time spent playing games per day should be evaluated in future research. Fourth, because the survey did not include data from before campus closure, it is not possible to assess teachers' internet use before mandatory online teaching began (e.g., time spent engaging in online activities and associated PIU levels). The present study, while attempting to collect data with a sample representative of the population of teachers in China (including teachers with minimal problematic internet use), did not include any items to assess teachers' amount of time spend in online gaming or social media use. Therefore, future research is encouraged to collect data on game and social media use experience in able to better interpret the findings. Consequently, the results of the present study may be affected by the fact that these factors were not controlled. Finally, as PNT of online teaching serves as a prominent source of stress during mandatory online teaching (9, 35), we hypothesized the role of this variable as an antecedent factor influencing the development of teachers' psychological distress and subsequently problems with PIU. However, we also acknowledge that teachers, as a group at high risk for mental illness, may experience other outcomes or contributing factors, in addition to PNT of online teaching, in relation to psychological distress. While distress may lead to other problems, in addition to PIU, during emergency situations like the pandemic, the limited scope of this study did not evaluate these issues. Therefore, other factors related to teachers' psychological distress, in addition to PNT of online teaching, and whether administrative support can alleviate teachers' psychological distress and common negative coping responses (e.g., drug and alcohol abuse) during mandatory online teaching, or similar conditions, should continue to be explored in future studies.

In conclusion, the present study found that schoolteachers in mainland China were likely to develop PIU because of the psychological distress caused by PNT of online teaching during the COVID-19 pandemic. Moreover, this PIU could further elevate psychological distress among schoolteachers. Additionally, the present study found that administrators' support was a significant protective factor for schoolteacher in not developing PIU. Therefore, healthcare providers and related stakeholders (e.g., governments and school managers) need to consider how to support teachers more effectively during times when online teaching is mandatory. This should help alleviate psychological distress and PIU issues among schoolteachers.

## Data availability statement

The raw data supporting the conclusions of this article will be made available by the authors, without undue reservation.

## Ethics statement

Electronic informed consent was obtained on the first page of the online survey, and the study was approved by Jianxi Psychological Consultant Association (IRB ref: JXSXL-2020-J013). Written informed consent for participation was not required for this study in accordance with the national legislation and the institutional requirements.

## Author contributions

I-HC and H-PC: conceptualization. I-HC, XL, and JG: methodology. X-MC and Y-TY: validation. H-PC and AP: formal analysis. XL, X-MC, and JG: investigation. I-HC and JG: resources. MG: writing—review and editing and supervision. I-HC: writing—original draft preparation and project administration. JG and C-YL: writing—review and editing. All authors read and approved the final manuscript.

## Funding

This research was supported by the 2022 Shandong Social Science Foundation Project Research on teaching management innovation in rural primary schools in the post-pandemic era (Project No.: 22CJYJ16).

## Conflict of interest

The authors declare that the research was conducted in the absence of any commercial or financial relationships that could be construed as a potential conflict of interest.

## Publisher's note

All claims expressed in this article are solely those of the authors and do not necessarily represent those of their affiliated organizations, or those of the publisher, the editors and the reviewers. Any product that may be evaluated in this article, or claim that may be made by its manufacturer, is not guaranteed or endorsed by the publisher.
