# Supplementary material for: Evaluating a cross-lagged panel model between problematic internet use and psychological distress and cross-level mediation of school administrator support on problematic internet use: The serial mediating role of psychological needs thwarting of online teaching and psychological distress
Source: Front Public Health. 2022 Nov 2;10:987366. doi: 10.3389/fpubh.2022.987366 (PMC9667893; doi:10.3389/fpubh.2022.987366)
Supplement: Supplementary file 1 [file Data_Sheet_1.docx]

**Supplementary files**

To evaluate possible attrition bias, we compared the differences between participants who were selected (*n* = 980) and who were not selected (*n* = 662) for the final sample of this study. The comparison was conducted using independent *t*-tests, which is a common method for detecting attrition bias (1) in terms of the variables of interest (administrators’ support, PNT of online teaching, psychological distress, PSMU, and PG). The results demonstrated only two significant differences between the two groups, which were for the variables of administrators’ support and PNT of online teaching; whereas there were no differences between groups for any other variables (i.e., psychological distress, PSMU, and PG at Time 1 and Time 2; see Supplementary Table S1). The effects were trivial for both of the two variables demonstrating a significant difference between the two groups. Therefore, considering there was no substantial difference between the two groups on the variables of interest, we deemed it reasonable to assume that sample attrition was not systematic and would not have an obvious effect on the results.

1. Miller RB, Wright DW. Detecting and Correcting Attrition Bias in Longitudinal Family Research. *Journal of Marriage and Family* (1995)57(4):921-929. doi:10.2307/353412

Supplementary Table S1 Comparison of samples selected or not selected in terms of the variables of interest

|  | Group | Mean (SD) | *t*-test (*p*-value) | Effect size: Cohen’s *d* (Interpretation) |
| --- | --- | --- | --- | --- |
| Administrators’ support | Participants selected as the final sample | 3.83 (0.67) | -2.69 (<0.01) | 0.14 (trivial effect) |
|  | Participants not selected as the final sample | 3.74 (0.65) |  |  |
| PNT of online teaching | Participants selected as the final sample | 40.31 (11.34) | 2.96 (<0.01) | 0.14 (trivial effect) |
|  | Participants not selected as the final sample | 42.01 (11.62) |  |  |
| Psychological distress– Measured at Time 1 | Participants selected as the final sample | 18.94 (18.92) | 1.74 (0.08) | 0.09 (trivial effect) |
|  | Participants not selected as the final sample | 20.68 (21.96) |  |  |
| Psychological distress– Measured at Time 2 | Participants selected as the final sample | 18.72 (21.69) | 0.92 (0.36) | 0.05 (trivial effect) |
|  | Participants not selected as the final sample | 19.73 (21.91) |  |  |
| PSMU– Measured at Time 1 | Participants selected as the final sample | 14.09 (4.74) | -0.06 (0.95) | 0.00 (trivial effect) |
|  | Participants not selected as the final sample | 14.07 (4.78) |  |  |
| PSMU– Measured at Time 2 | Participants selected as the final sample | 13.35 (4.55) | 0.19 (0.85) | 0.01 (trivial effect) |
|  | Participants not selected as the final sample | 13.39 (4.68) |  |  |
| PG– Measured at Time 1 | Participants selected as the final sample | 12.92 (5.49) | 0.85 (0.40) | 0.04 (trivial effect) |
|  | Participants not selected as the final sample | 13.16 (5.99) |  |  |
| PG– Measured at Time 2 | Participants selected as the final sample | 13.59 (6.22) | 1.03 (0.30) | 0.05 (trivial effect) |
|  | Participants not selected as the final sample | 13.93 (6.60) |  |  |

*n* of the participants selected as the final sample = 980, *n* of the participants not selected as the final sample = 662

PNT of online teaching: Psychological need thwarting of online teaching; PSMU: Problematic social media use; PG: Problematic gaming;


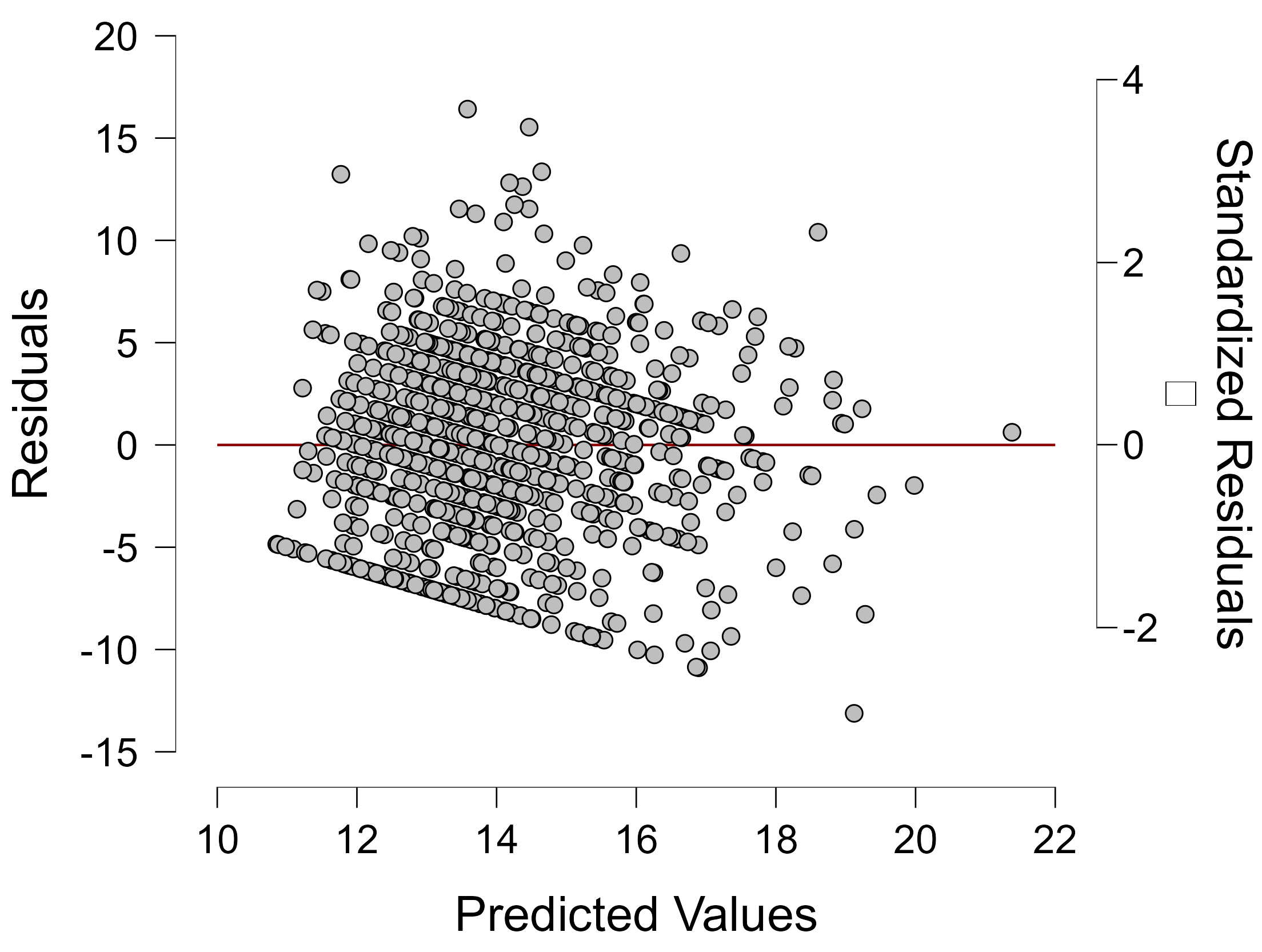


Supplementary Figure S1 Residual scatter plots for problematic social media use


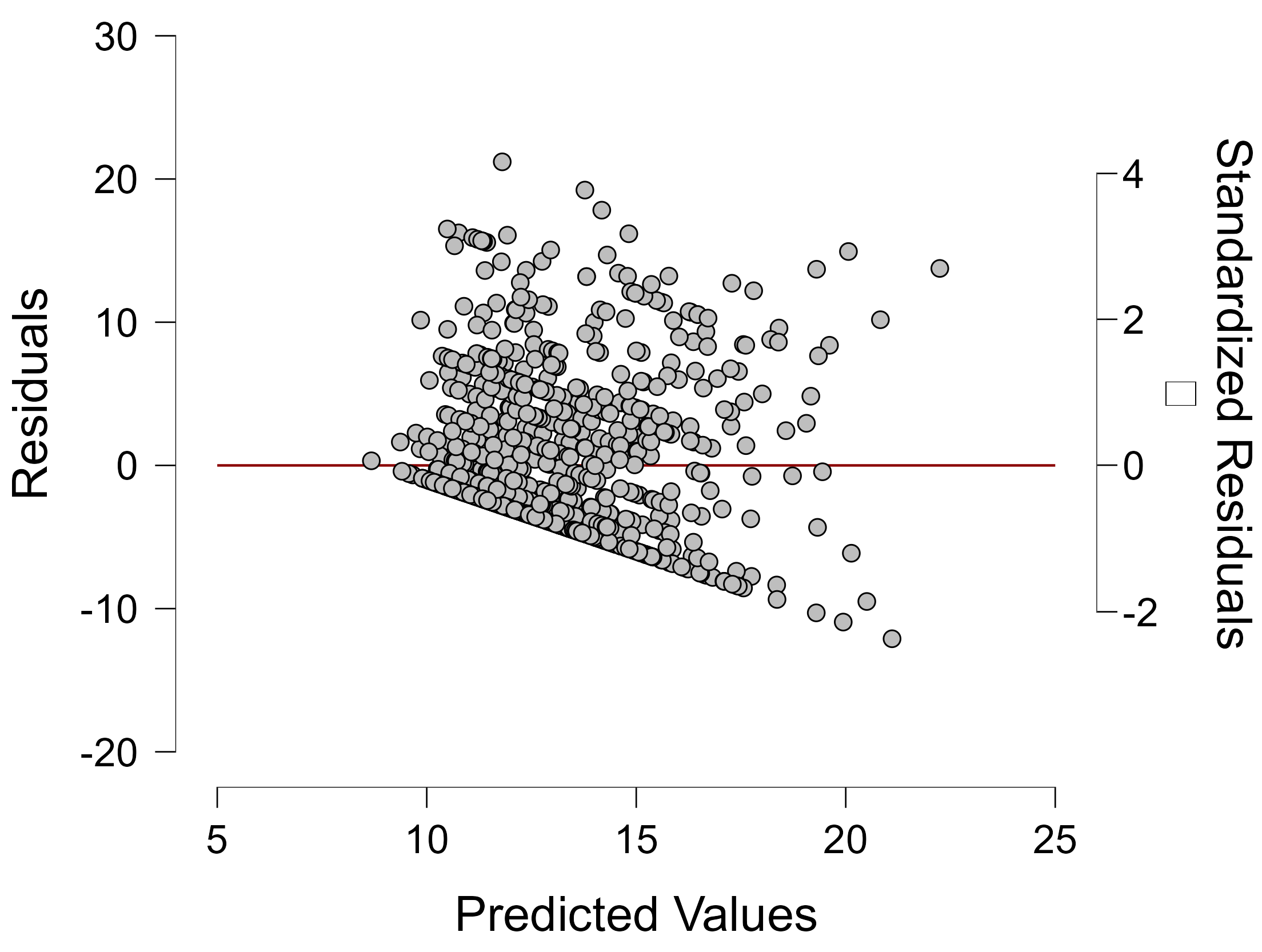


Supplementary Figure S2 Residual scatter plots for problematic gaming


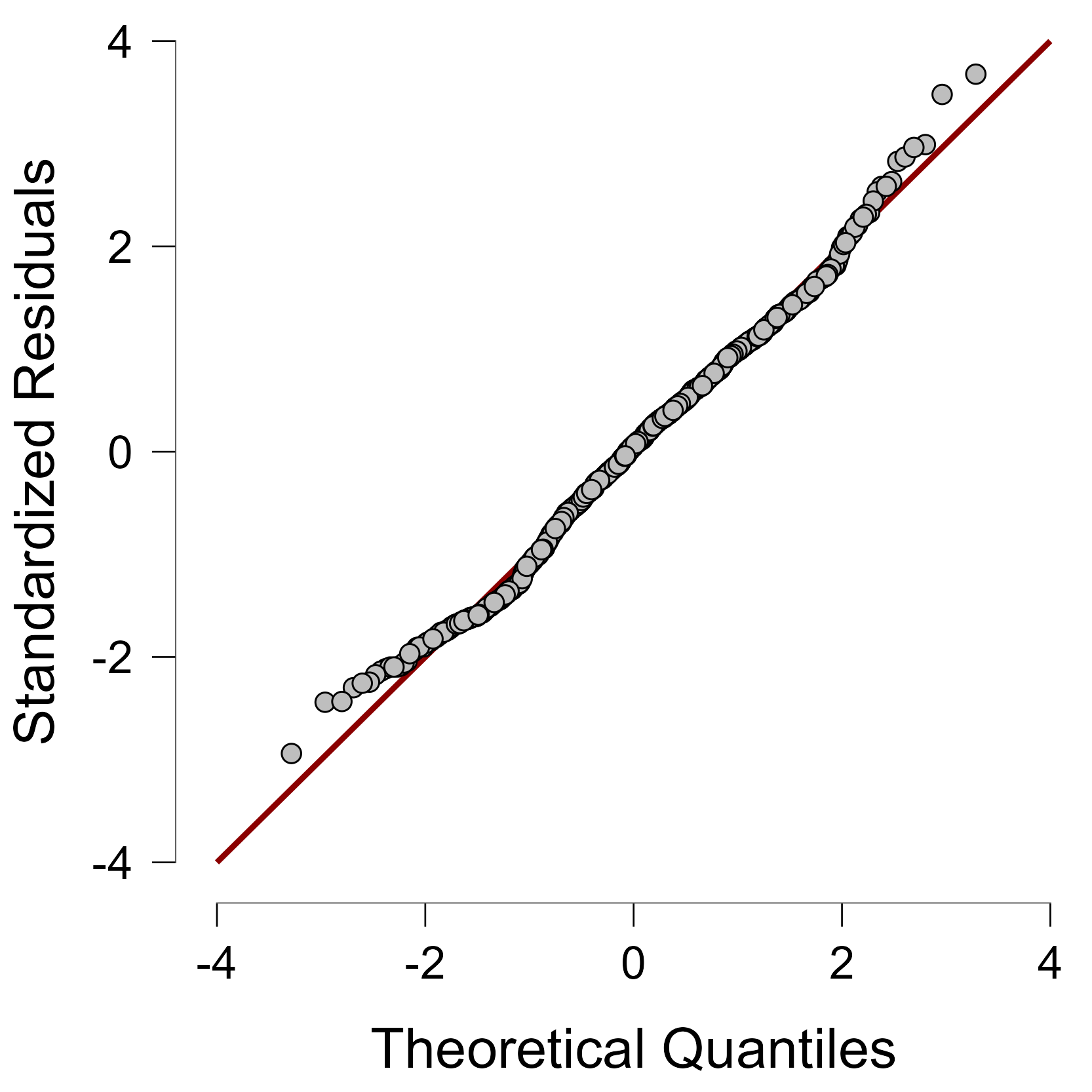


Supplementary Figure S3 Q–Q plot for problematic social media use


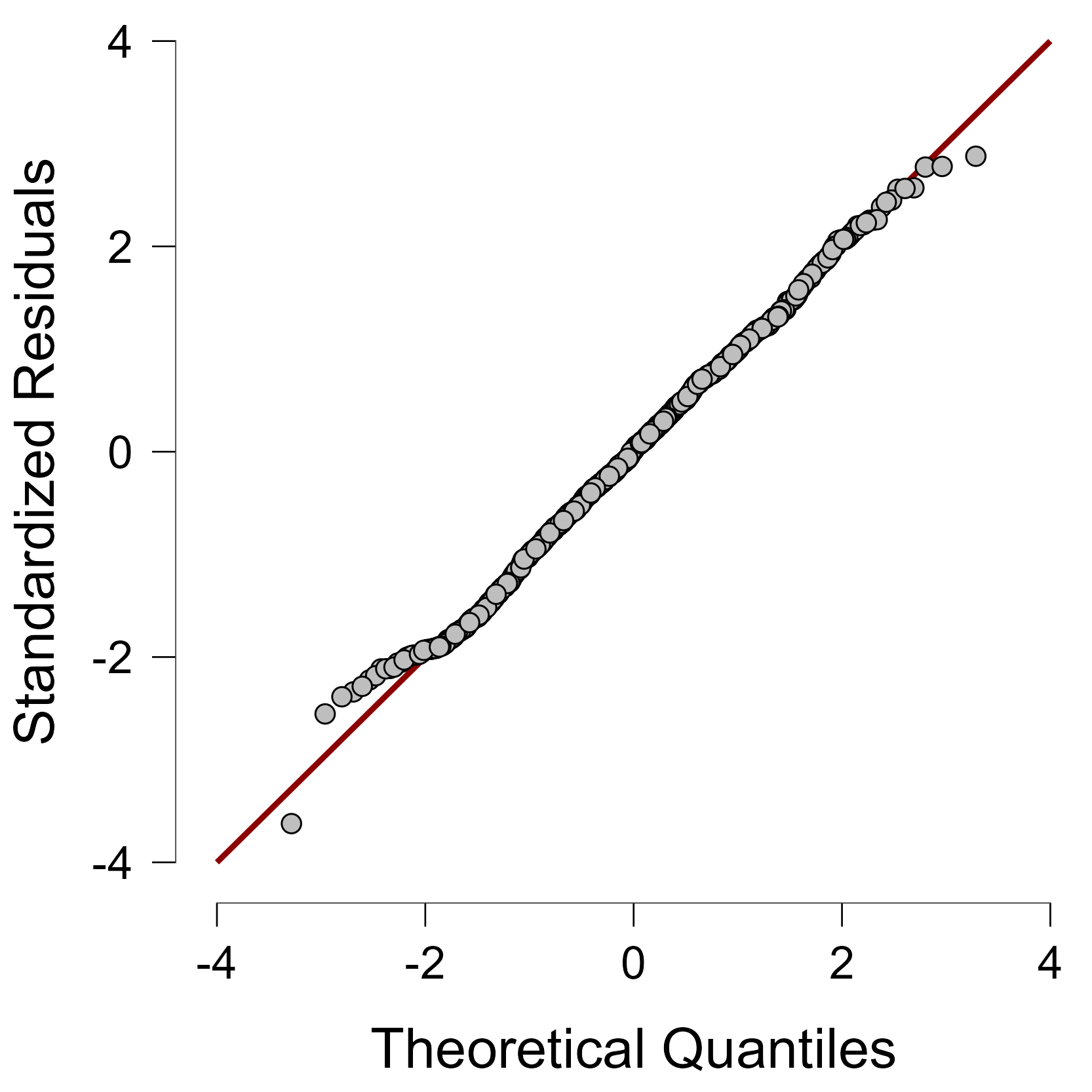


Supplementary Figure S4 Q–Q plot for problematic gaming
